# L-glutamine sensitizes Gram-positive-resistant bacteria to gentamicin killing

**DOI:** 10.1128/spectrum.01619-23

**Published:** 2023-10-26

**Authors:** Lvyuan Fan, Zhiyu Pan, Yilin Zhong, Juan Guo, Xu Liao, Rui Pang, Qingqiang Xu, Guozhu Ye, Yubin Su

**Affiliations:** 1 Department of Cell Biology & Institute of Biomedicine National Engineering Research Center of Genetic Medicine, MOE Key Laboratory of Tumor Molecular Biology, Guangdong Provincial Key Laboratory of Bioengineering Medicine, College of Life Science and Technology,Jinan University, Guangzhou, China; 2 Center for Excellence in Regional Atmospheric Environment and Key Laboratory of Urban Environment and Health, Institute of Urban Environment, Chinese Academy of Sciences, Xiamen, China; 3 Guangdong Provincial Key Laboratory of Microbial Safety and Health,State Key Laboratory of Applied Microbiology Southern China, Guangdong Institute of Microbiology, Guangdong Academy of Sciences, Guangzhou, China; University of Manitoba, Winnipeg, Manitoba, Canada

**Keywords:** methicillin-resistant *Staphylococcus aureus*, L-glutamine, gentamicin, ΔpH, membrane permeability, ROS

## Abstract

Because of the stubborn resistance to antibiotics, treating clinically resistant bacteria is a tricky business. Although considerable attention has been devoted to preventing and treating infections from drug-resistant bacteria, few studies are available on combining metabolites and antibiotics to combat methicillin-resistant *Staphylococcus aureus* (MRSA). This study found that exogenous L-glutamine potentiated aminoglycoside (gentamicin)-mediated killing efficacy in a dose- and time-dependent manner in MRSA (USA300 cell line) and other Gram-positive-resistant bacteria including MRSA 252, *Listeria monocytogenes*, and *Corynebacterium diphtheriae*. L-glutamine promoted the uptake of gentamicin through increasing the membrane permeability and was correlated with disrupted pH gradient. Furthermore, L-glutamine decreased intracellular reactive oxygen species by glutathione, which also increased USA300 sensitivity to gentamicin. We also demonstrated that combined treatment with gentamicin and L-glutamine enhanced the survival of MRSA-infected mice. In conclusion, we developed a promising therapeutic strategy for treating Gram-positive-resistant bacterial infections.

**IMPORTANCE:**

Methicillin-resistant *Staphylococcus aureus* (MRSA) infection severely threatens human health due to high morbidity and mortality; it is urgent to develop novel strategies to tackle this problem. Metabolites belong to antibiotic adjuvants which improve the effect of antibiotics. Despite reports of L-glutamine being applied in antibiotic adjuvant for Gram-negative bacteria, how L-glutamine affects antibiotics against Gram-positive-resistant bacteria is still unclear. In this study, L-glutamine increases the antibacterial effect of gentamicin on MRSA, and it links to membrane permeability and pH gradient (ΔpH), resulting in uptake of more gentamicin. Of great interest, reduced reactive oxygen species (ROS) by glutathione was found under L-glutamine treatment; USA300 becomes sensitive again to gentamicin. This study not only offers deep understanding on ΔpH and ROS on bacterial resistance but also provides potential treatment solutions for targeting MRSA infection.

## INTRODUCTION

Methicillin-resistant *Staphylococcus aureus* (MRSA) is a multidrug-resistant (MDR) Gram-positive bacteria, which is one of the most common and drug-resistant pathogens in hospital and community. The severity of invasive MRSA infections in the bloodstream, skin, and soft tissue causes high morbidity and mortality (1 – 3). The World Health Organization has labeled MRSA infection as a major global health problem. Therefore, novel approaches for combating bacterial resistance are urgently needed and underlying mechanisms of MDR Gram-positive bacteria must be understood.

Although antibiotic resistance is a major concern, drugs such as lipoglycopeptides, chloramphenicols, aminoglycosides, and rifamycins remain irreplaceable for combating most *S. aureus*-related diseases (4, 5). In particular, aminoglycosides such as gentamicin is combined with other antibiotics for treatment of MRSA infection (6, 7). The effectiveness of gentamicin against MRSA lies in its ability to disrupt protein biosynthesis and thus inhibit bacterial growth, but resistance to this drug is already increasing (8). Combining multiple antibiotics is a promising approach to combat MRSA and other drug-resistant bacteria (9 – 11), but resistance will eventually develop in response to novel drug combinations (12). Moreover, combination drug therapy has the potential side effect of increased toxicity for patients (12, 13). Therefore, new therapeutic approaches are needed that do not rely exclusively on antibiotics.

Promising evidence suggests that altering the metabolic state of bacteria is effective against drug resistance (14, 15). Our and other studies found that exogenous metabolites could reprogram bacterial metabolomes from resistant to sensitive phenotypes, restoring antibiotic susceptibility (16 – 24). Thus, the combination of an antibiotic and a non-antibacterial agent has been considered as an alternative therapeutic approach. In recent years, the metabolite L-glutamine could synergize with rifampicin against mycobacterial persisters via promoting energy metabolism (25); Zhao et al. found that it also promoted nucleoside biosynthesis in *Escherichia coli* and these nucleosides interacted with bacterial two-component system CpxA/CpxR and upregulated OmpF to exert metabolism-altering effects (21). Besides, L-glutamine increased the antibacterial effect of gentamicin to resistant *E. coli* (23). Still, it is unclear whether L-glutamine could synergistically interact with antibiotics against Gram-positive resistant bacteria, and the important mechanism is poorly understood.

In this study, we aimed to investigate synergistic antibiotic effects of L-glutamine and gentamicin on MRSA *in vitro* and *in vivo*, as well as explore the underlying mechanisms. The combination therapy described here may be a promising strategy for combating MRSA infections.

## RESULTS

### Exogenous L-glutamine promotes aminoglycosides to kill MRSA

**Fig 1:**
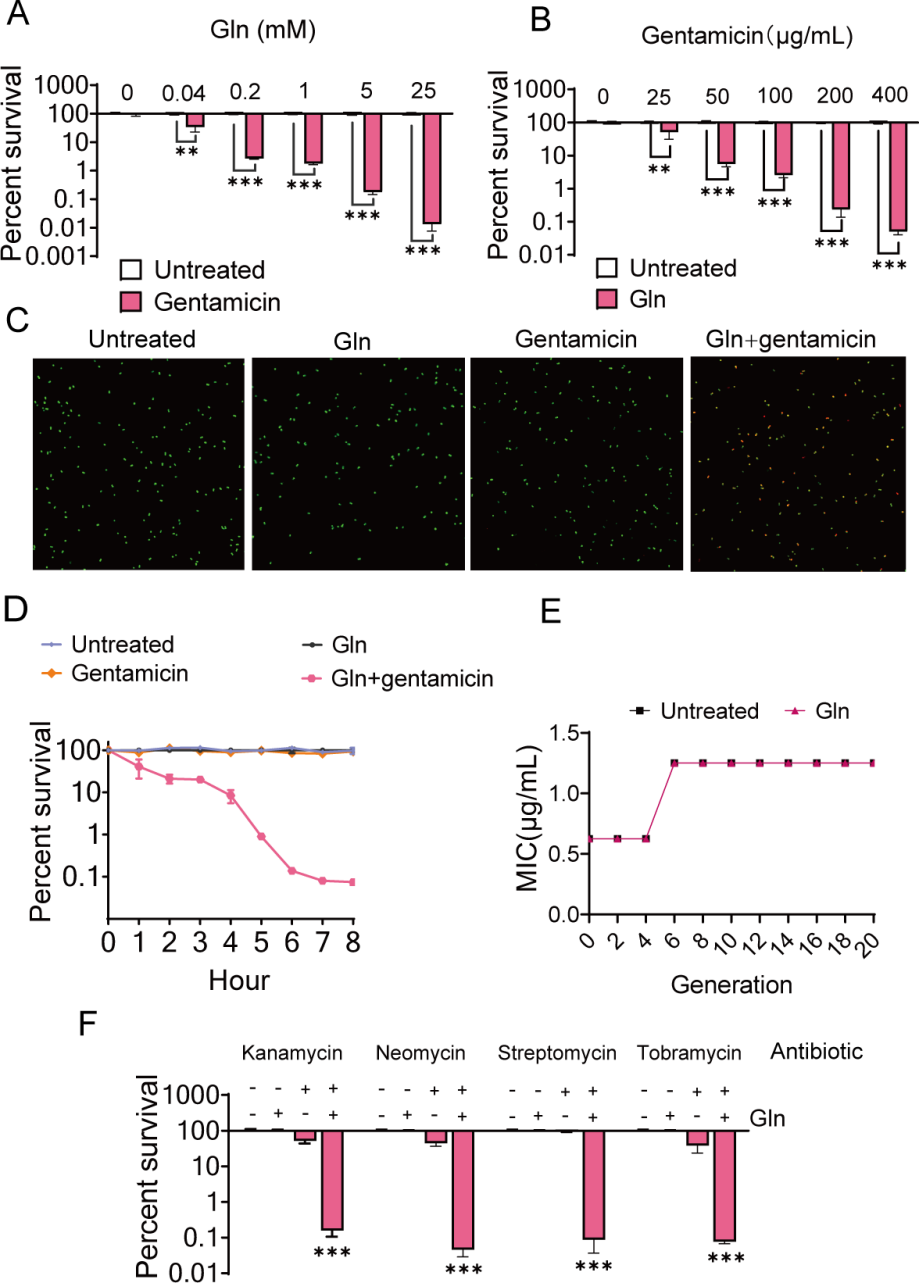
Effect of exogenous L-glutamine on the susceptibility of Gram-positive bacteria USA300 to aminoglycosides. (**A**) Percent survival of USA300 treated with different concentrations of L-glutamine (Gln) and/or gentamicin (200 µg/mL). (**B**) Percent survival of USA300 treated with different concentrations of gentamicin (200 µg/mL) and/or L-glutamine (5 mM). (**C**) Confocal micrographs of USA300 with different concentrations of PBS, L-glutamine, gentamicin, and their combination for 6 h stained by the Live & Dead Bacterial Staining Kit. Viable bacteria were stained green with DMAO, while dead bacteria were stained red with EthD-III. (**D**) The time effect of bactericidal efficacy with L-glutamine (5 mM) and/or gentamicin (200 µg/mL) treatment. (**E**) MIC of USA300, which was propagated in M9 medium with or without glutamine (5 mM) for twenty generations, and the concentration of gentamicin was 200 µg/mL. (**F**) Percent survival of USA300 treated with L-glutamine and other aminoglycosides, kanamycin (400 µg/mL), tobramycin (400 µg/mL), streptomycin (800 µg/mL), and neomycin (1.0 mg/mL). All data are displayed as mean ± SEM. *P < 0.05, **P < 0.01, and ***P < 0.001, determined by one-way ANOVA.

When in the present doses of exogenous L-glutamine, gentamicin exerted a dose-dependent inhibitory effect on the viability of USA300, a Gram-positive MRSA strain (Fig. 1A). Alone, 200 µg/mL gentamicin did not exhibit bactericidal activity against USA300 cells, but in combination with L-glutamine, the potentiated efficacy was over 500-fold. Additionally, the cell survival rate was decreased with an increasing dose of gentamicin plus 5 mM L-glutamine (Fig. 1B). Fluorescence microscopic imaging showed that the combination of L-glutamine and gentamicin resulted in enhanced red and orange fluorescence, whereas the monotreatment resulted in green fluorescence only (Fig. 1C). The killing effect was also time dependent, with the highest bactericidal activity effect at 6–8 h (Fig. 1D). To determine whether the bacteria developed resistance to L-glutamine and gentamicin-enabled potentiation, USA300 was propagated in M9 medium containing increasing concentrations of gentamicin with or without glutamine for 20 generations. Up to the 5th generation, the minimum inhibitory concentration (MIC) value remained unchanged for both gentamicin and L-glutamine plus gentamicin. From the 6th generation, the MIC value doubled but no longer increased with increasing generations (Fig. 1E). This suggests that the synergistic use of gentamicin and L-glutamine had a negligible effect on the reemergence of MRSA resistance. Highly synergistic effects of L-glutamine and other aminoglycosides (e.g., kanamycin, tobramycin, streptomycin, and neomycin) against USA300 were also observed (Fig. 1F). These data show that L-glutamine potentiates the bactericidal characteristics of aminoglycosides against Gram-positive-resistant bacteria.

### L-glutamine increases gentamicin uptake through disrupting ΔpH and increasing membrane permeability

**Fig 2:**
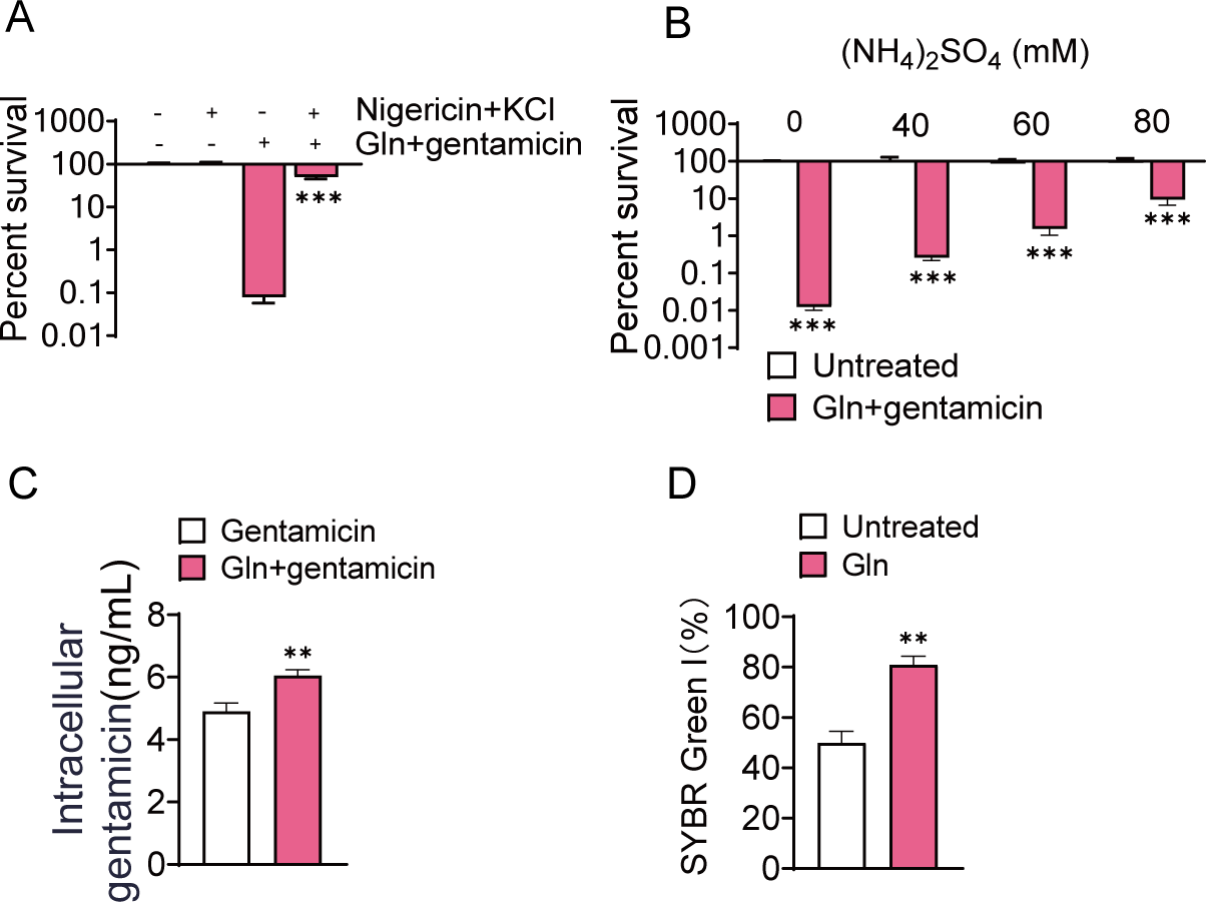
ΔpH and membrane permeability are involved in the synergistic bactericidal effect of L-glutamine and gentamicin against Gram-positive bacteria USA300. (**A**) Percent survival of USA300 treated with nigericin/excess K^+^ and/or the combined treatment with gentamicin (200 µg/mL) and L-glutamine (5 mM). (**B**) Percent survival of USA300 treated with different concentrations of (NH_4_)_2_SO_4_ and/or the combined treatment with gentamicin (200 µg/mL) and L-glutamine (5 mM). (**C**) Change in the intracellular gentamicin content under gentamicin treatment with or without L-glutamine. (**D**) Changes in the membrane permeability induced by glutamine treatment. Membrane permeability was detected by 1 × SYBR Green I. All data are displayed as mean ± SEM. *P < 0.05, **P < 0.01, and ***P < 0.001, determined by one-way ANOVA.

It has been reported that exogenous metabolites could improve antibiotic activity through altering pH gradient (ΔpH) (26, 27). For example, nigericin/excess K^+^ decreased the intracellular pH, which was responsible for the loss of potentiating activity for sodium acetate in the combination ceftazidime against *Pseudomonas aeruginosa* biofilms (27). We found that nigericin/excess K^+^ abolished the synergistic effect of L-glutamine and gentamicin against USA300, similarly (Fig. 2A). Additionally, (NH_4_)_2_SO_4_ eliminated the potentiating bactericidal effect of L-glutamine and gentamicin (Fig. 2B), possibly through triggering decreases of intracellular pH (26). The results suggest that the disappearance of the synergistic effect resulted from the disrupted ΔpH by nigericin/excess K^+^ and (NH_4_)_2_SO_4_. Besides, we also observed increased gentamicin uptake by USA300 cells after L-glutamine treatment (Fig. 2C). The research showed that an alteration in ΔpH elevates gentamicin uptake (26, 28, 29), which was also demonstrated in our study. Since antibiotics absorption was attributable to alteration of the membrane permeability, we measured permeability in USA300 by SYBR Green I and demonstrated a clear increase after treatment with L-glutamine (Fig. 2D and Fig. S1). These results indicate that L-glutamine promotes gentamicin uptake via disrupting ΔpH and increasing membrane permeability.

### L-glutamine-induced decreases in ROS levels are involved in the bactericidal effect

**Fig 3:**
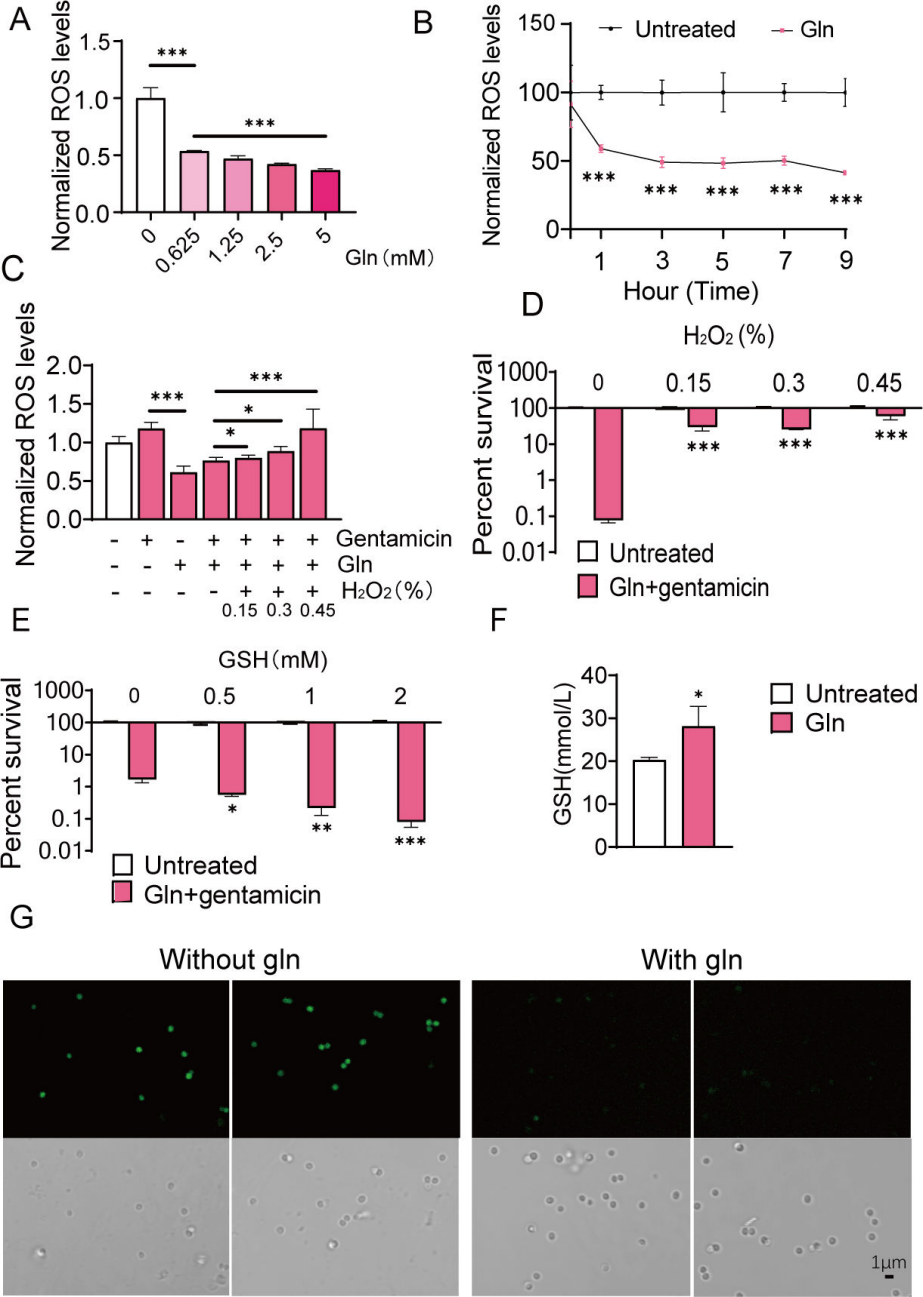
ROS is involved in the synergistic bactericidal effect of L-glutamine and gentamicin. (**A**) Changes in ROS levels induced by different concentrations of L-glutamine. (**B**) ROS levels induced by L-glutamine for time-gradient incubation. (**C**) Gentamicin and/or L-glutamine (5 mM) treatments induced changes in ROS levels. (**D**) The role of H_2_O_2_ in the synergistic bactericidal effect of L-glutamine (5 mM) and gentamicin (200 µg/mL). (**E**) The role of GSH in the synergistic bactericidal effect of L-glutamine (1.25 mM) and gentamicin (200 µg/mL). (**F**) The level of GSH under L-glutamine (5 mM) treatments. (**G**) Bright-field and corresponding fluorescence images of USA300 under L-glutamine (5 mM) treatment stained by DCFH-DA. All data are displayed as mean ± SEM. *P < 0.05, **P < 0.01, and ***P < 0.001, determined by one-way ANOVA.

It has been proven that antibiotic effectiveness is associated with reactive oxygen species (ROS) production and exogenous metabolites have been found to influence ROS levels (22). Here, we observed that L-glutamine treatment could decrease ROS in a dose- dependent manner (Fig. 3A) and generate a lower level of ROS in the 9th hour (Fig. 3B) and the ROS fluorescence signal was significantly weakened after L-glutamine treatment by fluorescence detection (Fig. 3G). We next examined the effect of H_2_O_2_ (contributor to ROS) on the bactericidal effect of L-glutamine plus gentamicin. Addition of gentamicin alone could increase ROS levels, and H_2_O_2_ restored the ROS content with the increase of concentration when combining L-glutamine with gentamicin (Fig. 3C). Furthermore, adding a dose of H_2_O_2_ abolished the bactericidal effects of L-glutamine and gentamicin (Fig. 3D). Conversely, GSH (eliminator to ROS) could improve gentamicin and L-glutamine to kill UAS300 (Fig. 3E). In addition, more GSH was produced under L-glutamine treatment (Fig. 3F). Thus, L-glutamine-induced decreases by GSH in intracellular ROS levels contribute to gentamicin sensitivity of USA300 cells.

### L-glutamine improves gentamicin efficacy *in vitro* and *in vivo*

**Fig 4:**
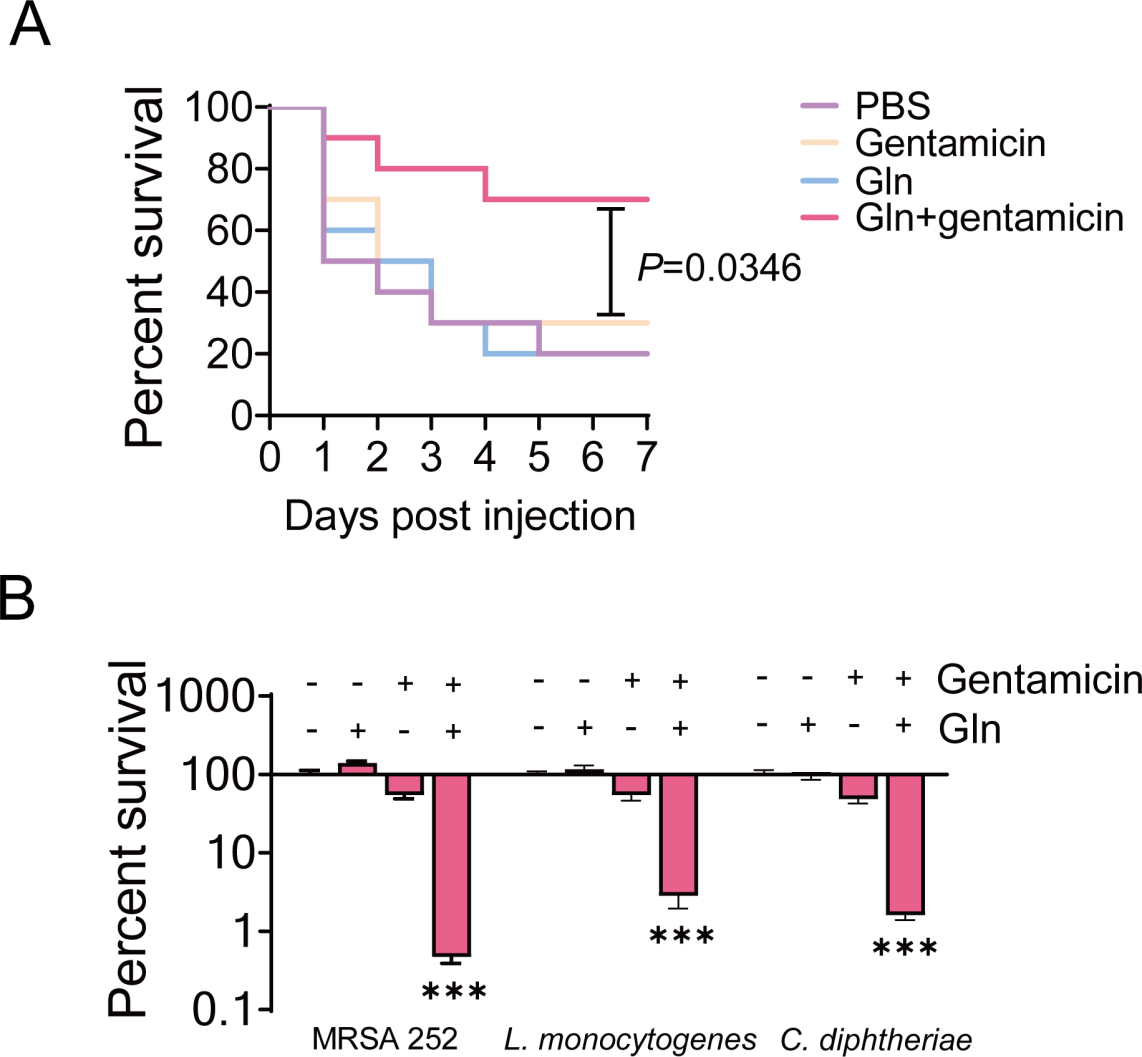
L-Glutamine improves the antimicrobial efficacy of gentamicin *in vitro* and *in vivo*. (**A**) The synergistic bactericidal effects of combined treatment with L-glutamine (5 mM) and gentamicin against MRSA252, *L. monocytogenes*, and *C. diphtheriae*, with gentamicin concentrations of 400 µg/ mL, 800 µg/mL, and 4 µg/mL, respectively. (**B**) Percent survival of MRSA-infected mouse in the presence of L-glutamine and/or gentamicin. All data are displayed as mean ± SEM. *P < 0.05, **P < 0.01, and ***P < 0.001, determined by one-way ANOVA.

Previous studies have demonstrated the synergistic bactericidal effects of L-glutamine with antibiotics against Gram-negative bacteria *in vitro*. Here, we demonstrated the potentiated efficacy against other Gram-positive drug-resistant bacteria, such as MRSA 252, *Corynebacterium diphtheria*, and *Listeria monocytogenes* (Fig. 4A). Subsequently, we evaluated the synergistic effects of L-glutamine and gentamicin *in vivo*. First, BALB/c mice were infected with USA300 (1 × 10^9^ CFU) via tail vein injection and then treated with a single dose of gentamicin and/or L-glutamine via intraperitoneal injection. Percent survival of MRSA-infected mice was not significantly improved after a single treatment with gentamicin or glutamine. However, combining gentamicin and L-glutamine caused mouse survival to reach 70% (Fig. 4B), which had similar good effect to the positive treatment with vancomycin (100 mg kg^−1^) alone (Fig. S2).These results suggest that combined treatment enhances antimicrobial activity against Gram-positive bacteria, thus improving survival rates in MRSA-infected mice.

## DISCUSSION

The synergistic bactericidal activity of metabolites and antibiotics has been widely studied (30 – 32). Metabolites, such as amino acids, are typical adjuvants for antibiotics. For example, combined alanine and gentamicin treatment elevated bactericidal efficacy against antibiotic-resistant *Vibrio alginolyticus* (33). Moreover, glycine, serine, and threonine potentiated kanamycin-mediated killing of *Edwardsiella piscicida* (17). Recently, glutamine promoted ampicillin to eliminate multidrug-resistant uropathogenic *E. coli* (21). L-glutamine is a special amino acid with two NH_4_^+^. Current researches of L-glutamine mainly focus on its relation with cancer, given that glutamine is the metabolic fuel supporting enhanced tumor proliferation, invasion, and bioenergetics (34, 35). However, few studies are available on the role of L-glutamine in restoring antibiotic susceptibility of Gram-positive resistant bacteria.

**Fig 5:**
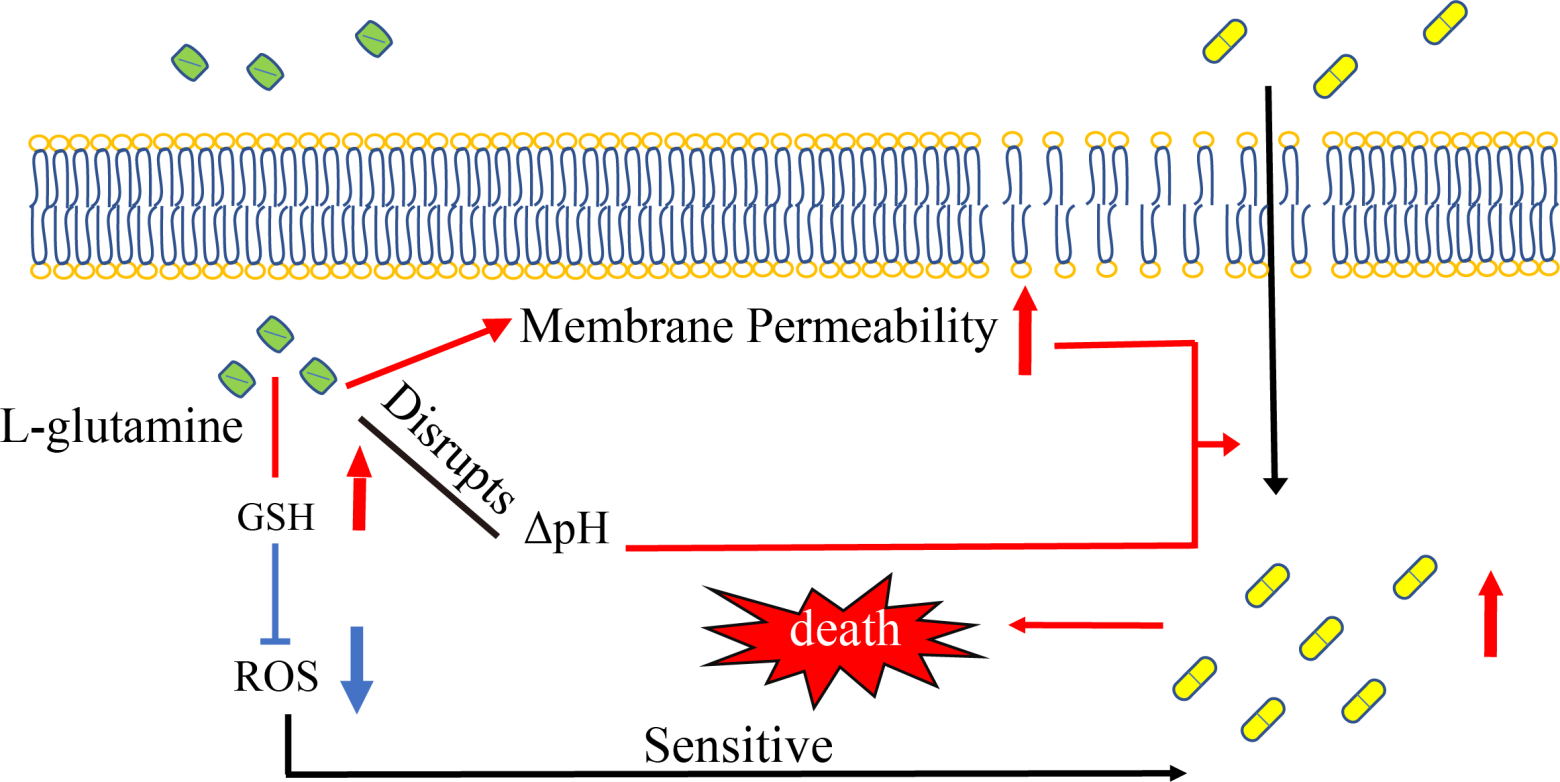
Proposed mechanism involved in the synergistic bactericidal effects of L-glutamine and gentamicin against MRSA. The treatment of L-glutamine disrupted the ΔpH and increased cell membrane permeability, which boosted the uptake of gentamicin and is resultant of bacterial death. Meanwhile, L-glutamine treatment decreased ROS levels by GSH, which promoted the sensitivity of MRSA to gentamicin.

In this study, we found strong synergistic bactericidal effects of gentamicin and L-glutamine against MRSA. The combined treatment altered ΔpH, cell membrane permeability, and ROS levels (Fig. 5). Multiple studies have demonstrated that pH plays an important role in bacterial resistance. For instance, ΔpH mediated aminoglycoside uptake (36), and alkaline conditions increased antibiotic efficacy (37). Thus, pH imbalance may contribute to antibiotic resistance via inducing changes in the antibiotic efflux systems and proton motive force (28, 38, 39). Nigericin/excess K^+^ or (NH_4_)_2_SO_4_ was used to disrupt ΔpH. Besides, our findings also revealed that exogenous L-glutamine increased USA300 cell membrane permeability. Similarly, previous studies have reported that L-lysine improves membrane permeability of bacterial species such as *Acinetobacter baumannii*, *E. coli*, and *Klebsiella pneumoniae* (26). The results showed ΔpH and membrane permeability did influence the synergistic effect of L-glutamine and gentamicin. Therefore, L-glutamine induced a change of ΔpH and an increase of cell membrane permeability elevated gentamicin uptake, resulting in greater bactericidal effect.

Surprisingly, the presence of L-glutamine eliminated a portion of gentamicin-induced ROS. L-glutamine is an important biosynthetic component of some antioxidants, such as glutathione (40). Our results showed glutathione was increased which metabolized from L-glutamine. In fact, the role of ROS in antibiotic tolerance and resistance is complex, and there are some controversies and challenges (41, 42), being deleterious or beneficial depending on the conditions (43 – 45). For example, ROS induced by non-lethal antibiotics could contribute to the development of antibiotic resistance (46 – 48). Moreover, an increase in ROS levels lowered MRSA sensitivity to *Amomum villosum* Lour essential oil (49). Another study found that N-acetylcysteine lowered wild-type mutagenesis through decreasing intracellular ROS levels, in turn hampering the development of antibiotic resistance (50). These studies suggest that limiting intracellular ROS levels helps suppress antibiotic resistance in pathogenic bacteria. Our study found that L-glutamine treatment decreased ROS levels implying that the metabolite contributes to the synergistic bactericidal activity of L-glutamine and gentamicin.

In conclusion, L-glutamine increased gentamicin efficacy against Gram-positive bacteria and improved the survival rate of mice infected with MRSA. Further mechanistic studies indicated that L-glutamine enhanced aminoglycoside uptake through disrupting ΔpH, increasing cell membrane permeability and decreasing ROS. However, the influence of L-glutamine on ROS requires further research. Our findings suggest that L-glutamine had antioxidant characteristics that benefited the synergistic bactericidal activity of L-glutamine and gentamicin.

## MATERIALS AND METHODS

### Bacterial strains, growth conditions, and chemical agents

Methicillin-resistant *S. aureus* USA300_FPR3757 was kindly provided by Dr. Hua Zhou, from Zhejiang University. MRSA 252, *Listeria monocytogenes*, and *Corynebacterium diphtheriae* were kindly provided by Dr. Haixin Chen, from Shenzhen Bay Lab. These strains were cultured in 30 mL Luria-Bertani (LB) broth (HuanKai Microbiology Technology Co. Ltd., Guangdong, China) at 37°C and 220 rpm for 12 h. L-Glutamine and gentamicin (Aladdin, China), other antibiotics (Macklin, China), Live & Dead Bacterial Staining Kit (YESEN, China), 2′,7′-dichlorofluorescein diacetate (DCFH-DA, Sigma, USA), nigericin (APExBIO), NH_4_Cl and (NH_4_)_2_SO_4_ (Macklin, China), SYBR Green I (Biosharp, China), and glutathione (GSH) (Biosharp, China) were used in this study.

### Bactericidal study

Overnight bacterial cultures were collected and adjusted to an optical density of 0.2 at 600 nm (OD_600_) with M9 medium (containing 10 mM acetate, 1 mM MgSO_4_, and 100 µM CaCl_2_) and incubated in a constant-temperature incubator at 37°C and 220 rpm for 6 h. Bacteria were treated with L-glutamine, antibiotics, and/or other chemical agents in the M9 medium. Finally, 100 µL of the culture was serially diluted. An aliquot (10 µL) of each dilution was plated onto LB agar to determine the bacterial count and calculated CFU/mL (14).

### Assessment of drug resistance development

Based on the method reported by Zhao et al. (21), bacteria were cultured in LB medium overnight, collected by centrifugation at 8,000 rpm for 5 min, and washed three times with sterile saline. The bacteria were resuspended in M9 medium and diluted to OD_600_ = 1.0. Next, 100 µL of the bacterial suspension (5 × 10^7^ CFU) was added to each well of a 96-well microplate. Additionally, 100 µL of a series of twofold dilutions of gentamicin or gentamicin plus 5 mM glutamine diluted in M9 medium was added to each well. The mixture was then incubated at 37°C. The minimum inhibitory concentration was determined after 16 h based on the lowest concentration of antimicrobial agent that visually inhibited the growth of microorganisms. The surviving bacteria on LB agar were recultured, and the MIC was determined repeatedly for 20 generations. Three biological replicates were used for each experiment.

### Measurement of ROS

The intracellular ROS in the USA300 strain was measured using 2′,7′-dichlorodihydrofluorescein diacetate (DCFH-DA, Sigma, USA) (51). Overnight bacterial cultures were collected and adjusted to OD_600_ = 0.6 with M9 medium and incubated in a constant-temperature incubator at 37°C and 220 rpm for 6 h with gentamicin and/or L-glutamine. A mixture containing 194 µL of bacterial cultures and 4 µL of 2′,7′-dichlorodihydrofluorescein diacetate (200 µL, final concentration 20 µM) was incubated in microplates at 37°C for 1 h in the dark. Fluorescence was immediately measured at an excitation wavelength of 485 nm and emission wavelength of 515 nm using a plate reader (CLARIO Star Plus, Germany).

Laser scanning confocal microscopy was used to determine the fluorescence of DCFH-DA. Overnight bacterial cultures were collected and adjusted to OD_600_ = 0.2 with M9 medium and incubated in a constant-temperature incubator at 37°C and 220 rpm for 6 h with or without L-glutamine. A mixture including 98 µL of bacterial cultures and 2 µL of 2′,7′-dichlorodihydrofluorescein diacetate (100 µL, final concentration 20 µM) was incubated in microplates at 37°C for 1 h in the dark and observed using 35-mm laser scanning confocal microscopy at the wavelength of 488 nm (OLYMPUS, Japan).

### Measurement of GSH

Overnight bacterial cultures were collected and adjusted to OD_600_ = 1.0 with M9 medium and incubated in a constant-temperature incubator at 37°C and 220 rpm for 6 h with or without L-glutamine. A total of 10 mL of the cultures was collected to assess the concentration of GSH using a GSH test kit (Nanjing Jiancheng Bioengineering Institute, China). All tests were performed in duplicate.

### Measurement of membrane permeability

Overnight bacterial cultures were collected and adjusted to OD_600_ = 0.2 with M9 medium and incubated in a constant-temperature incubator at 37°C and 220 rpm for 6 h with or without L-glutamine. A total of 100 µL of the culture was obtained and diluted 10-fold and then stained with 1 × SYBR Green I for 15 min. Green (FTIC: absorption wavelength/emission wavelength = 488 nm/520 nm) fluorescence intensity was determined using a flow cytometer (the total number of cells was up to 10,000), and the threshold of FSC and SSC was 8,000 (Beckman Coulter, Brea, CA, USA) (52). All tests were performed in duplicate, and the positivity rate of the stained bacteria was evaluated.

### Determination of intracellular gentamicin concentration

Overnight bacterial cultures were collected and adjusted to OD_600_ = 1.0 with M9 medium and incubated in a constant-temperature incubator at 37°C and 220 rpm for 6 h with or without L-glutamine. Bacterial cultures were harvested, washed three times with PBS, and then lysed by sonication (200 W total power with 60% output, 2 s pulse, and 3 s pause) for 40 min on ice. The supernatant was collected by centrifugation at 12,000 rpm for 10 min at 4°C. Finally, gentamicin concentration was assessed using a gentamicin ELISA rapid test kit (Cusabio, Hubei, China) (53).

### *In vivo* synergy in the mouse infection model

To evaluate the efficacy of the drugs against MRSA strains *in vivo*, BALB/c mice were used. Overnight bacterial cultures were washed, collected by centrifugation at 8,000 rpm for 5 min, and adjusted to a final density of 1 × 10^9^ CFU/mL with PBS. After 1 week of adaptation, BALB/c mice (8–10 weeks) were inoculated with a total of 100 µL 1 × 10^9^ CFU/mL MRSA (USA300) bacterial suspension in PBS (20 g/mouse) by tail vein injection. After 6 h, mice were randomly divided into five groups (*n* = 10 per group) and intraperitoneally injected with 200 µL PBS (200 µL), gentamicin (5 mg kg^−1^), glutamine (100 mg kg^−1^), gentamicin (5 mg kg^−1^) + glutamine (100 mg kg^−1^), or vancomycin (100 mg kg^−1^) twice a day for a week. The mice were observed, and the observations were recorded daily. The log-rank test was used to analyze the survival of mice.

## Data Availability

Data will be made available upon request.

## SUPPLEMENTAL MATERIAL

Supplemental file 1 spectrum.01619-23-s0001.docx Legends to Fig. S1 and S2.

Supplemental file 2 spectrum.01619-23-s0002.tif Fig. S1 (Changes in the membrane permeability induced by L-glutamine treatment).

Supplemental file 3 spectrum.01619-23-s0003.tif Fig. S2 (Percent survival of MRSA-infected mouse in the presence of vancomycin [100 mg/kg]).

ASM does not own the copyrights to Supplemental Material that may be linked to, or accessed through, an article. The authors have granted ASM a non-exclusive, world-wide license to publish the Supplemental Material files. Please contact the corresponding author directly for reuse.
