# Supplementary material for: L-glutamine sensitizes Gram-positive-resistant bacteria to gentamicin killing
Source: Microbiol Spectr. 2023 Oct 26;11(6):e01619-23. doi: 10.1128/spectrum.01619-23 (PMC10715002; doi:10.1128/spectrum.01619-23)
Supplement: Supplemental file 1 — Legends to Fig. S1 and S2. [file spectrum.01619-23-s0001.docx]

**Supplementary FIG 1** Changes in the membrane permeability induced by L-glutamine treatment. SYBR Green I was used to value the permeability of cell membranes by detecting FTIC. Flow cytometry result showed different rate of being stained within 15 min under the treatment of L-glutamine. The representative staining in three independent experiments are provided.

**Supplementary FIG 2** Percent survival of MRSA-infected mouse in the presence of vancomycin (100 mg/kg). All data are displayed as mean ± SEM. *P* < 0.05, determined by one-way ANOVA.
