# Supplementary figures and images for: L-glutamine sensitizes Gram-positive-resistant bacteria to gentamicin killing
Source: Microbiol Spectr. 2023 Oct 26;11(6):e01619-23. doi: 10.1128/spectrum.01619-23 (PMC10715002; doi:10.1128/spectrum.01619-23)

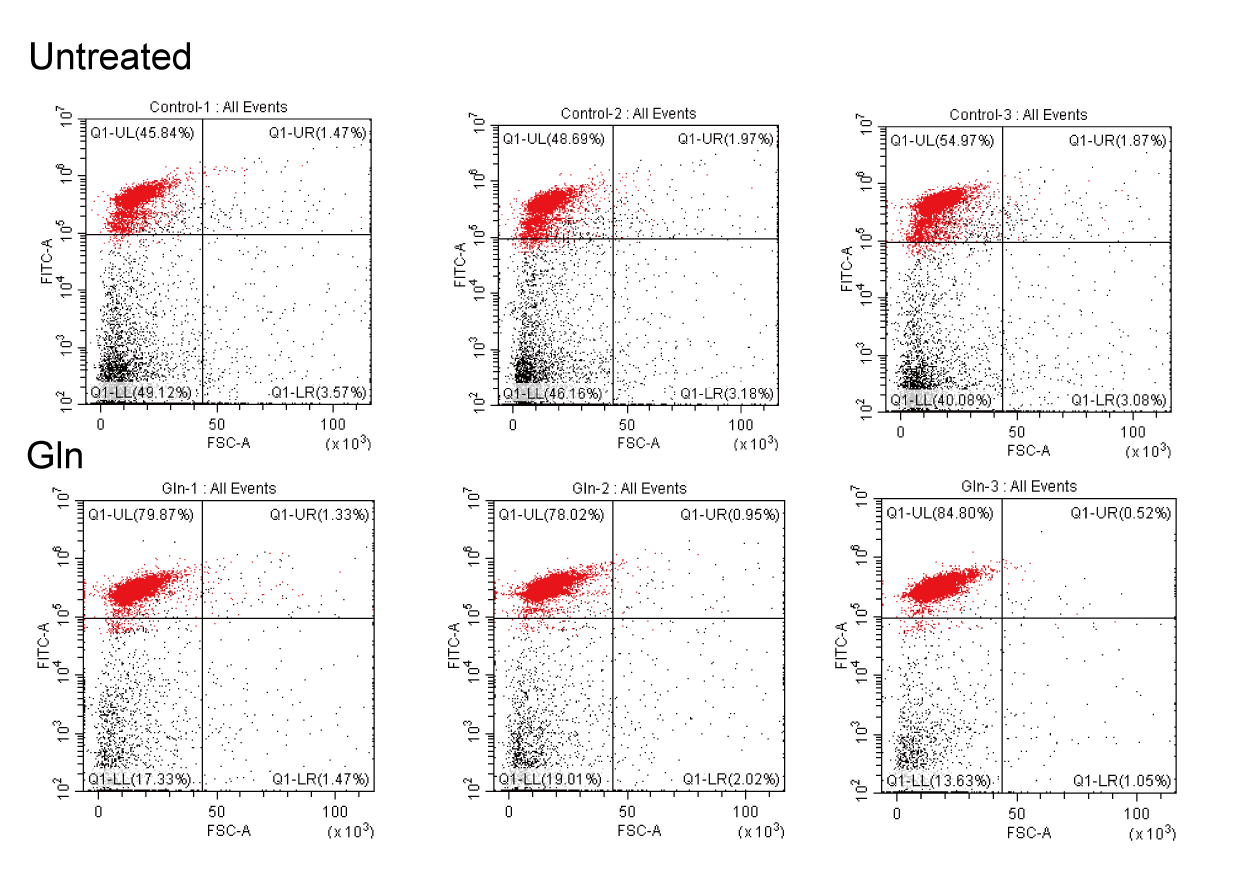

Supplement: Supplemental file 2 — Fig. S1 (Changes in the membrane permeability induced by L-glutamine treatment). [file spectrum.01619-23-s0002.tif]

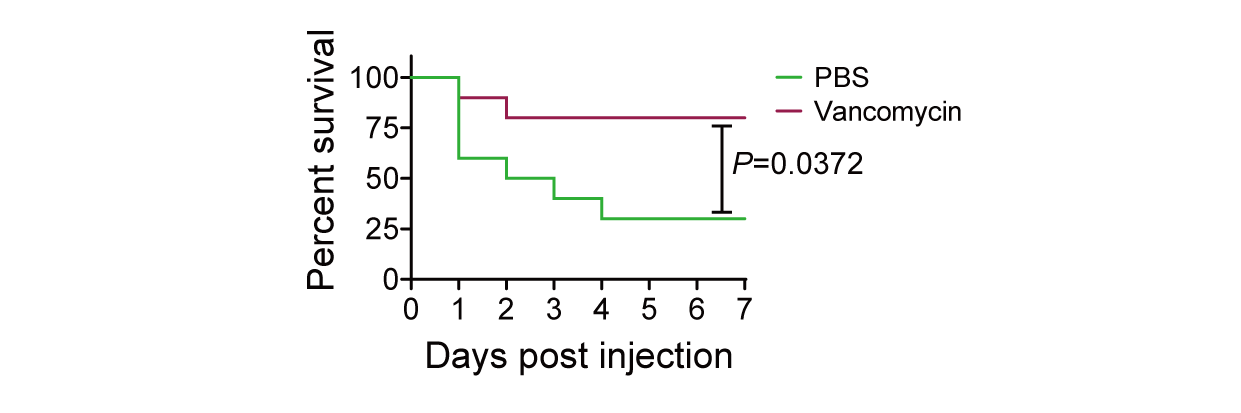

Supplement: Supplemental file 3 — Fig. S2 (Percent survival of MRSA-infected mouse in the presence of vancomycin [100 mg/kg]). [file spectrum.01619-23-s0003.tif]
